# Efficiency estimation of intrapleural and thoracic paravertebral block in combination with general anesthesia at thoracoscopic interventions

**DOI:** 10.1186/cc10940

**Published:** 2012-03-20

**Authors:** J Sabirov, A Khadjibaev, V Sharipova

**Affiliations:** 1 Republican Reseach Centre of Emegency Medicine, Tashkent, Uzbekistan

## Introduction

Chest injuries and traumas have become one of the most common reasons for admitting patients to emergency surgical hospitals in recent years.

## Methods

Ninety patients admitted to the RRCEM urgently with chest traumatic injuries have been examined. They were divided into two groups against the applied method of anesthesia. First (control) group (47 patients, 38.5 ± 2.4 years): IPA was done before the induction of anesthesia into the second intercostal space from the damaged side with bupivakain at a dose of 75 to 100 mg. Analgesic component maintained by the abovementioned IPA and phentanyl bolus dosing. The second group (43 patients, 36.8 ± 5.4 years): one-sided TPVB maintained before the induction at ThIV, ThVII levels 0.5% - 5 ml (25 mg) bupivakain dosing (at the average total 75 to 100 mg) with posterior paravertebral area catheterization. Analgesic component maintained by paravertebral analgesia and phentanyl bolus dosing.

## Results

The differences in hemodynamics indexes appeared at the traumatic moment of operation. In the group using IPA, medium hypertension with ABP rise in 25.5%, higher rate of HR in 26.1% and GPVR in 22% were observed and were followed by the decrease of SV on 24.6% and EF on 13% compared with the second group. Conducting anesthesia in the first group, hyperdynamic reactions of the systemic hemodynamics at the separate traumatic levels of operation were followed by unbalance of hemodynamic rhythms indicating insufficient prevention from surgical aggression. In the second group, as the result of development of segmental sympathetic block the indexes of ABP, HR and GPVR were not higher than normal.

## Conclusion

Both methods of regional anesthesia cut short pain syndrome sufficiently and safely in patients with chest injuries before an operative intervention. Introduction of the TPVB component into the anesthesia scheme of thoracoscopic operative interventions allows one to provide additional antinociceptive protection in the intraoperative period with minimal stress of central and peripheral parameters and promotes the reduction of narcotic analgesic use due to significant analgesic efficiency and neurovegetative protection.

